# Supplementary material for: The gut commensal Blautia maintains colonic mucus function under low-fiber consumption through secretion of short-chain fatty acids
Source: Nat Commun. 2024 Apr 25;15:3502. doi: 10.1038/s41467-024-47594-w (PMC11045866; doi:10.1038/s41467-024-47594-w)
Supplement: Supplementary file 1 — Supplementary Information [file 41467_2024_47594_MOESM1_ESM.pdf]

Supplementary Figure 1

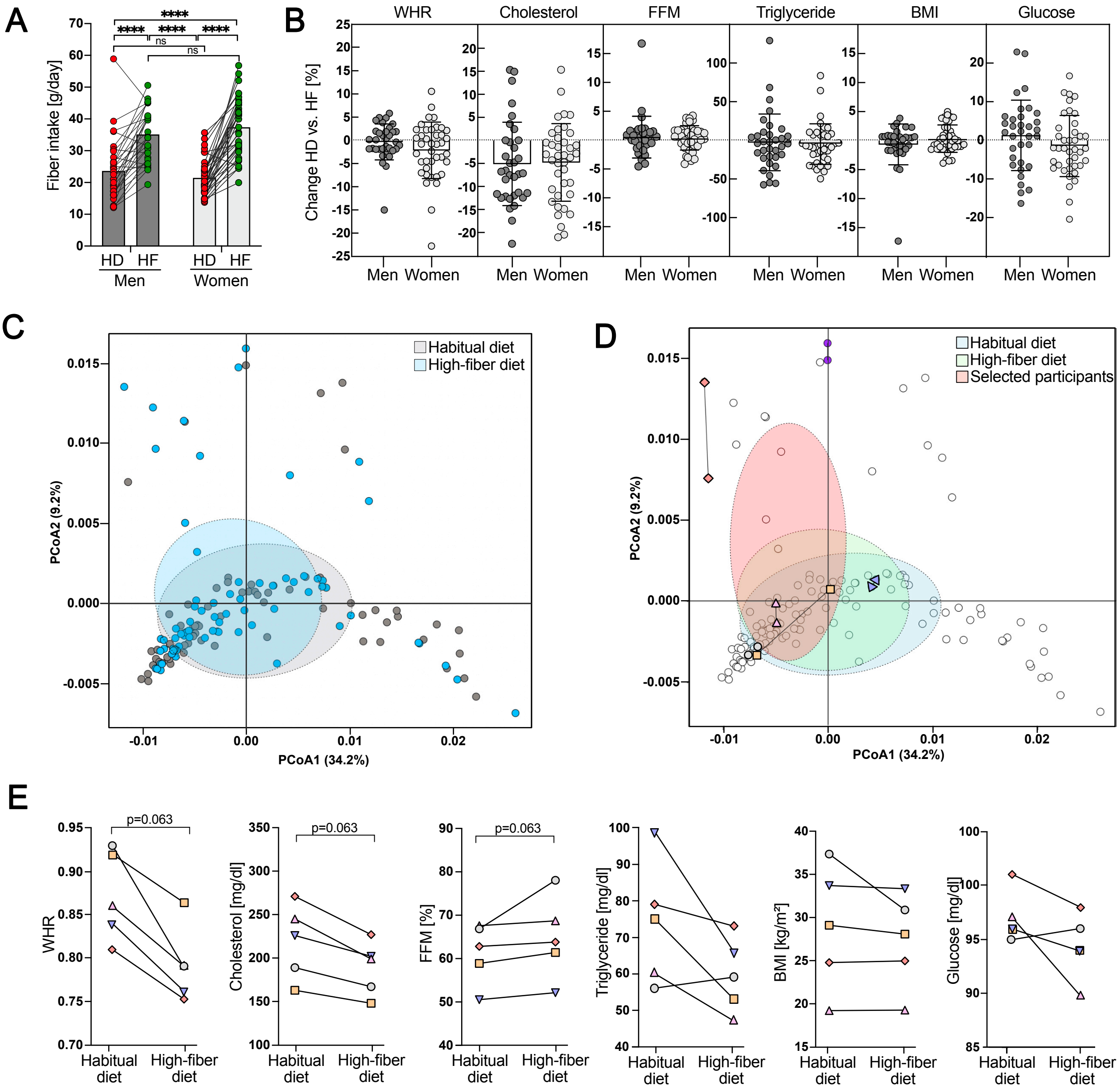

Supplementary Figure 1: (A) Fiber intake of male and female human participants (n=67) during habitual diet (HD) and after 12 weeks of high fiber (HF) intervention; (B) Change in metabolomic parameters for men and women after HF intervention. WHR=waist-to-hip ratio, FFR= free fat mass, BMI= body-mass index; (C) Weighted UniFrac of human participants on their HD and after the HF intervention; (D) Participants selected for microbiota transplantations are highlighted in coloured symbols and indicated with a red cloud. Please note that the donor marked as a purple circle was only included in the first FMT experiment (Fig 1) while the donor marked as an orange diamond was only included in the second FMT experiment (Fig 3); (E) Metabolic parameters of the 5 individuals selected as donors for FMT; Normal distribution of the data was tested using D'Agostino-Pearson test (A, B, E). Statistical significance was tested using 2-way ANOVA with Tukey's multiple comparison test (A), an unpaired t-test for normally distributed data and Mann-Whitney test for non-normally distributed data (B), PERMANOVA with 999 permutations (C, D) and Wilcoxon matched-pairs signed rank test (E) with p<0.0001(****) considered statistically significant. All P-values are two-sided. Linked to Figure 1. Source data are provided as a Source Data file.

Supplementary Figure 2

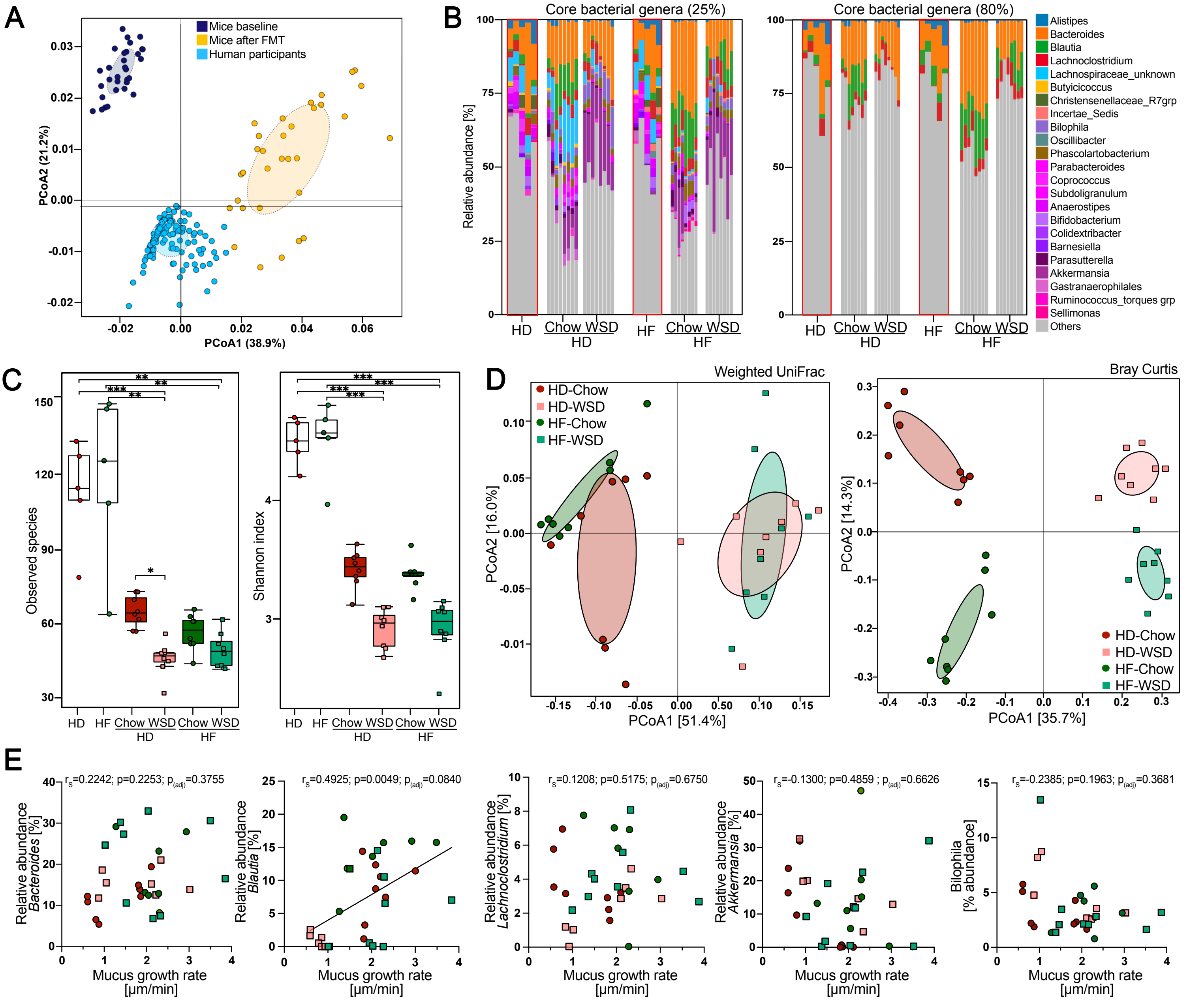

Supplementary Figure 2: (A) Weighted UniFrac PCoA of fecal bacteria from human study participants[32] as well as mice before (baseline) and after (termination) human-to-mouse FMT; (B) Core bacterial genera relative abundance plots for human donors (highlighted with red margin: HD= habitual diet, HF= high-fiber diet) and human microbiota-transplanted mice fed a chow or WSD. Core bacterial genera was defined as the bacterial genera present in 25% (left) or 80% (right) of all human study participants and transplanted mouse samples. (C) Stool bacterial alpha diversity of the human donors and transplanted mice, measured by observed species and Shannon diversity index. Kruskal-Wallis test was used to test for statistical significance; (D) Bacterial beta-diversity, measured by Weighted UniFrac distance matrix and Bray-Curtis dissimilarity matrix, of the transplanted mice. Statistical significance was tested by PERMANOVA with 999 permutations; (E) Spearman correlation analysis between mucus growth rate in the mouse distal colon and relative abundance of selected genera; P<0.05 (*) and p<0.01 (**) are considered statistically significant. All P-values are two-sided. Linked to Figure 2. Source data are provided as a Source Data file.

Supplementary Figure 3

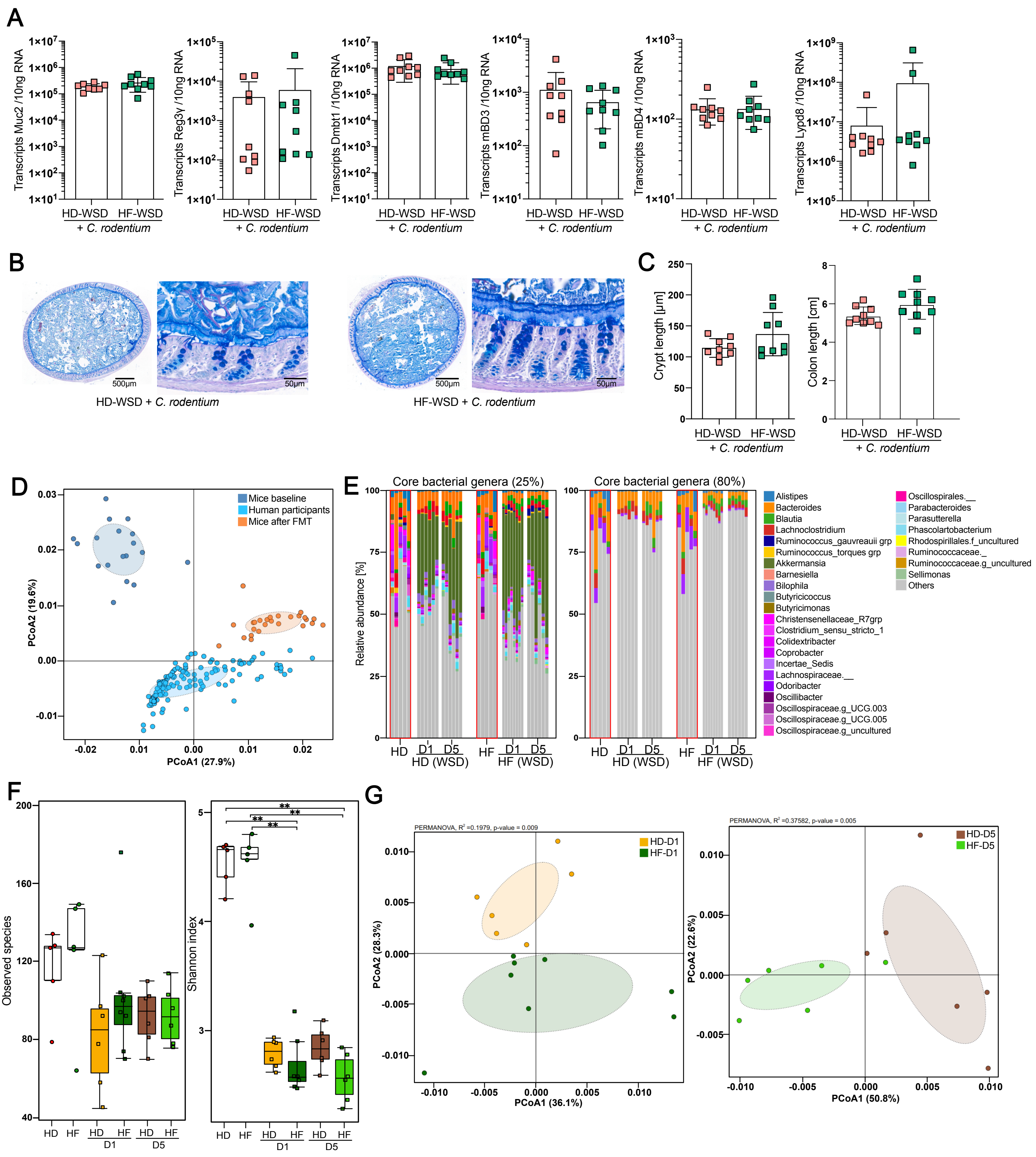

Supplementary Figure 3: (A) Absolute quantification of host defense protein/peptide transcripts in the distal colon of mice transplanted with human fecal microbiota and infected with *Citrobacter rodentium.* Human donors consumed their habitual diet (HD) or a high-fiber diet (HF) while mice were fed a WSD; (B) AB/PAS staining of distal colon sections from transplanted and infected mice. Representative images from 9 mice/group are shown, wherein at least 1 section/mouse was imaged and at least 10 crypts/mouse counted. Scale bars = 500 µm (full cross-section) and 50 µm (mucosal magnification); (C) Average colon length and crypt length in the distal colon of transplanted and infected mice; (D) Weighted UniFrac PCoA of bacterial communities from human study participants[32] and mice before (baseline) and after (termination) FMT+*C. rodentium* infection; (E) Core bacterial genera relative abundance plots for human donors (highlighted with red margin: HD= habitual diet; HF= high-fiber diet) and mice transplanted with the human microbiota and infected with *C. rodentium.* (D1=Day 1 post infection; D5=Day 5 post infection). Core bacterial genera was defined as the bacterial genera that were present in 25% (left) or 80% (right) of all human study participants and the transplanted mouse samples; (F) Stool bacterial alpha diversity of the human donors and mice transplanted with the human HD and HF samples at D1 and D5 post *C. rodentium* infection, measured by observed species and Shannon diversity index; (G) Weighted UniFrac PCoA of stool bacterial genera in the transplanted mice; Normal distribution of the data in A and C was tested with the D'Agostino-Pearson test and statistical significance was tested with an unpaired t-test (normally distributed data) or Mann-Whitney test (non-normally distributed data). Statistical differences in D and G were calculated with PERMANOVA and 999 permutations while Kruskal-Wallis test was used for (F). p<0.05 (*), p<0.01 (**) and p<0.001 (***) are considered statistically significant. All P-values are two-sided. Linked to Figure 3. Source data are provided as a Source Data file.

Supplementary Figure 4

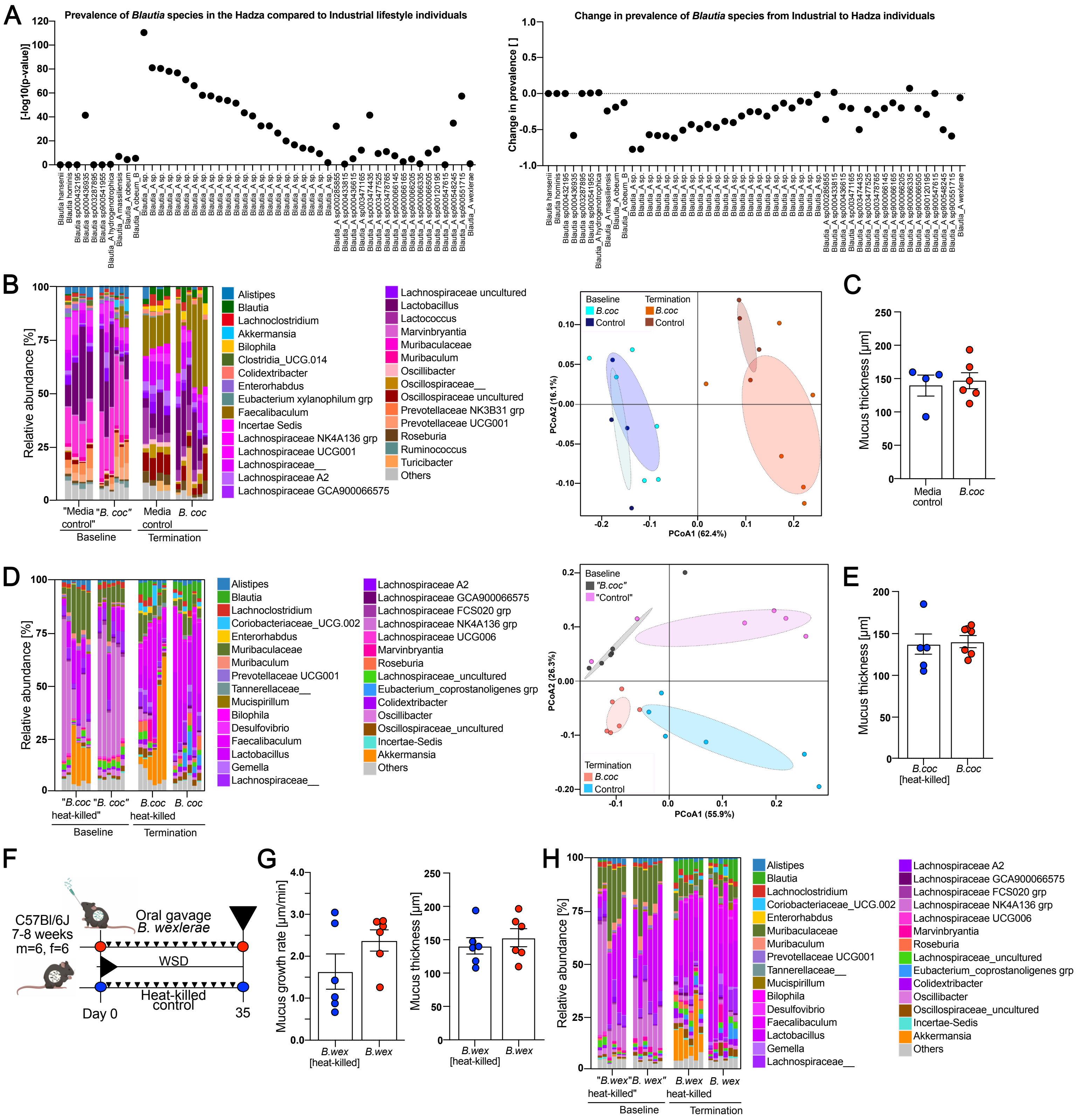

Supplementary Figure 4: (A) Log transform of Fisher's exact test p-values, comparing prevalence of the species in the Hadza, modern hunter-gatherers living in Tanzia, to industrial lifestyle individuals (negative value indicates greater prevalence in Hadza). Original data has been extracted from Meier et al.[37] and is graphically summarized here; (B) Relative abundance of top 30 microbial genera and Weighted UniFrac PCA before (baseline) and after (termination) supplementing WSD-fed mice with growth media control or *B. coccoides* in drinking water for 33 days (n=4 mice and n=6 mice, respectively); (C) Colonic mucus thickness of mice supplemented with *B. coccoides* through drinking water; (D) Relative abundance of top 30 bacterial genera and Weighted UniFrac PCoA before (baseline) and after (termination) supplementing WSD-fed mice with viable or heat-killed *B. coccoides* through oral gavage for 35 days (n=6 mice/group); (E) Colonic mucus thickness of mice supplemented with live or heat-killed *B. coccoides* through oral gavage; (F) Schematic representation of *B. wexlerae* supplementation through oral gavage to WSD-fed mice. Mice were supplemented with viable or heat-killed *B. wexlerae* (n=6 mice/group) through oral gavage for a period of 35 days, whereupon mucus function was investigated; (G) Mucus growth rate and mucus thickness of inner colonic mucus layer; (H) Relative abundance of top 30 bacterial genera before (baseline) and after (termination) supplementing mice fed a WSD with viable or heat-killed *B. wexlerae* through oral gavage for 35 days (n=6 mice/group); Normal distribution of the data in C, E and G was tested with the D'Agostino-Pearson test and statistical significance was determined using an unpaired t-test (normally distributed data) or Mann-Whitney U test (non-normally distributed data). Statistical differences in the Weighted UniFrac PCoA in B and D were calculated with PERMANOVA and 999 permutations. $p<0.05$ (*), $p<0.01$ (**) and $p<0.001$ (***) are considered statistically significant. All P-values are two-sided. Linked to Figure 4. Source data are provided as a Source Data file.

Supplementary Figure 5

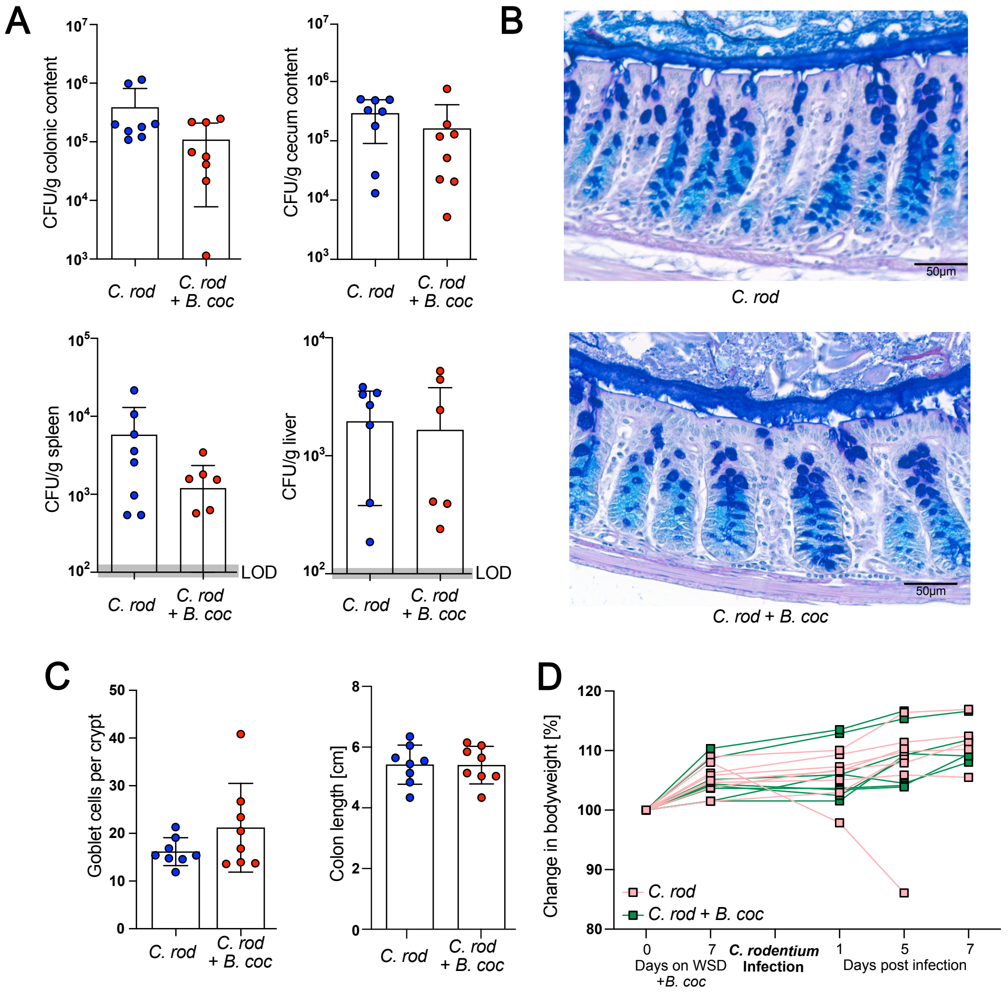

Supplementary Figure 5: (A) CFUs of *Citrobacter rodentium* in colonic content, cecum content, spleen and liver of mice 7 days after infection, and with or without supplementation of *B. coccoides* (LOD = limit of detection). (B) AB/PAS staining of distal colon sections from mice infected with *C. rodentium*, with or without supplementation of *B. coccoides*. Representative images from 8 mice/group are shown, wherein at least 1 section/mouse was imaged and at least 10 crypts/mouse counted. Scale bars = 50µm. (C) Average number of goblet cells per crypt and colon length in the infected mice. (D) Change in bodyweight before and after *C. rodentium* infection (n = 5-8mice/ group). Data in A and C are presented as mean ± SD. Normal distribution of the data in A, C and D was tested with the D'Agostino-Pearson test and statistical significance was determined using an unpaired t-test (normally distributed data) or Mann-Whitney U test (non-normally distributed data), with $p< 0.05$ (*) considered statistically significant. All P-values are two-sided. Linked to Figure 5. Source data are provided as a Source Data file.

Supplementary Figure 6

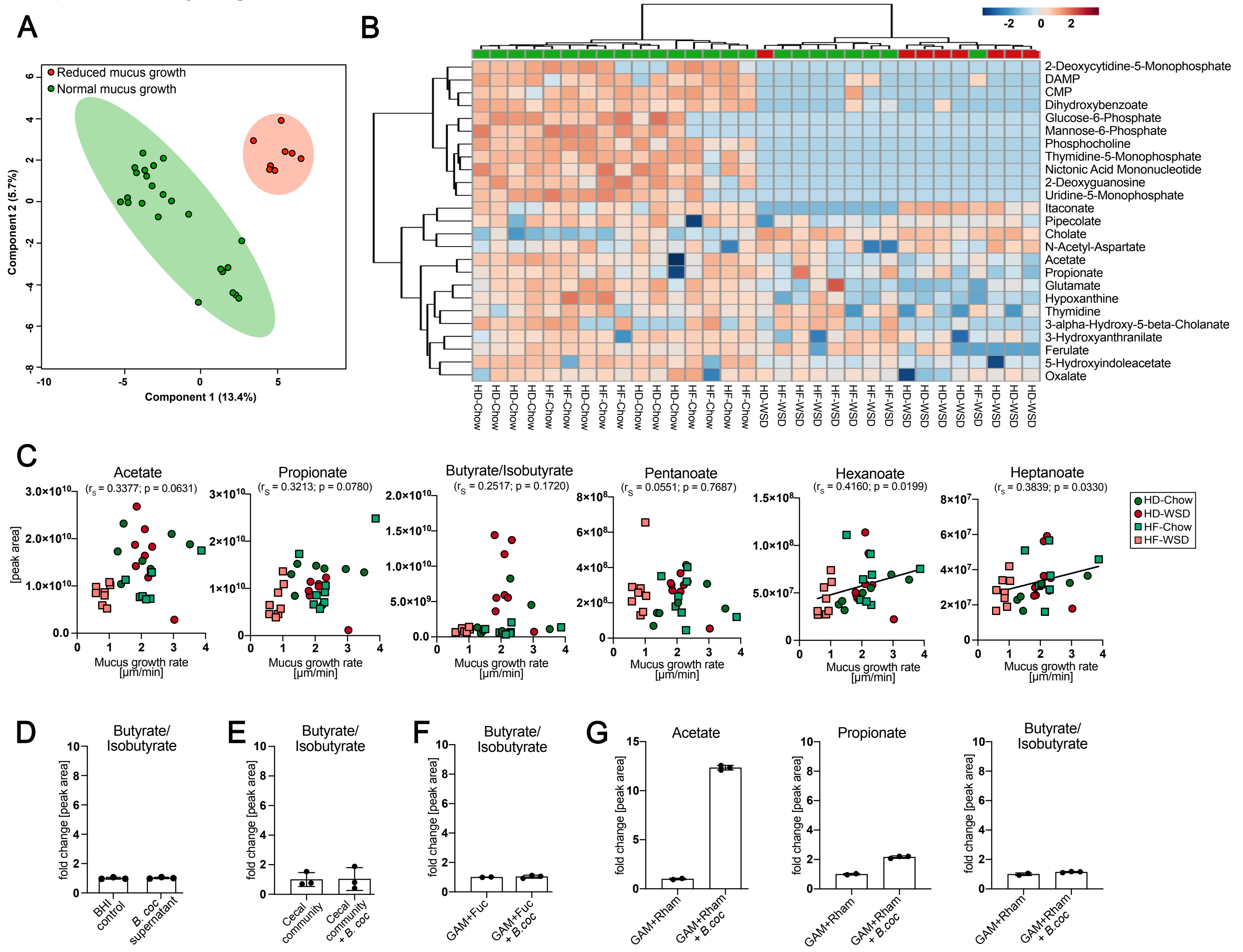

Supplementary Figure 6: (A) PLS-DA of high-throughput global metabolomics profiling of cecal content metabolites. Mouse groups (n=8 mice/ group) with average mucus growth rate (HD-Chow, HF-Chow and HF-WSD) are colored in green (n=24) while the mouse group with reduced mucus growth rate (HD-WSD) is colored in red (n=8); (B) Unsupervised hierarchical cluster analysis using Euclidian distance measurement of the 25 most altered metabolites between mucus phenotypes. Color scale indicates fold-change; (C) Correlation analysis between mucus growth rate in the distal colon and peak intensity of SCFAs, hexanoate and heptanoate, in the cecum of mice transplanted with the human microbiota. Group-specific colour/form coding as in Figure 1; (D) Quantification of butyrate/isobutyrate from supernatant of a 24h *B. coccoides* culture, supernatant of a 24h cecal community supplemented with *B. coccoides* (E) and supernatant of a 48h B. coccoides culture incubated in GAM media in the presence of fucose (F); (G) Quantification of acetate, propionate and butyrate/isobutyrate from supernatant of a 48h *B. coccoides* culture incubated in GAM media in the presence of rhamnose; Normal distribution of the data was tested with the D'Agostino Pearson test and data are presented as mean ± SD. Statistical significance between the two groups was determined by Mann-Whitney U test (D) while Spearman correlation analysis was used in (C), with p<0.05 (*) considered statistically significant. All P-values are two-sided. Linked to Figure 6. Source data are provided as a Source Data file.
