# Supplementary material for: The gut commensal Blautia maintains colonic mucus function under low-fiber consumption through secretion of short-chain fatty acids
Source: Nat Commun. 2024 Apr 25;15:3502. doi: 10.1038/s41467-024-47594-w (PMC11045866; doi:10.1038/s41467-024-47594-w)
Supplement: Supplementary file 3 — Description of Additional Supplementary Files [file 41467_2024_47594_MOESM3_ESM.docx]

**Description of Additional Supplementary Files**

**File Name:** Supplementary Data 1 **Description:** Amplicon sequence variants (ASVs) sequences assigned to *Blautia* (left) and the potential species, based on blast search (right).

**File Name:** Supplementary Data 2

**Description:** Correlations between bacterial genera and mucus growth rate of WSD-fed mice transplanted with human high-fiber driven microbiota or human habitual diet driven microbiota.
