# Supplementary material for: The gut commensal Blautia maintains colonic mucus function under low-fiber consumption through secretion of short-chain fatty acids
Source: Nat Commun. 2024 Apr 25;15:3502. doi: 10.1038/s41467-024-47594-w (PMC11045866; doi:10.1038/s41467-024-47594-w)
Supplement: Supplementary file 4 — Supplementary Data 1 [file 41467_2024_47594_MOESM4_ESM.pdf]

Supplementary Table 1

ASV	Potential species
215eab43672099dab90607033e9446f8	*Blautia coccoides, Blautia pseudococcoides, Blautia hominis, Blautia hansenii, Blautia marasmi*
224b0773460ed546fadcf609add6a4c9	*Blautia coccoides, Blautia pseudococcoides, Blautia hominis, Blautia hansenii*
64644dbd2c4d66c6dee31dd8a85a98ee	*Blautia coccoides, Blautia pseudococcoides, Blautia hominis, Blautia hansenii, Blautia marasmi*
ba11f2b6a318ccd52353476debe4febb	*Blautia coccoides, Blautia hansenii, Blautia producta*
25cf8a8d6aa065f5a5331a1a53db4ba0	*Blautia coccoides, Blautia hansenii, Blautia producta*
5f371e174ca1193fd938a9ec347c5d48	*Blautia intestinalis, Blautia sp.* strain Marseille
a638c4c026ba6f86f8a31fdefd11d281	*Blautia wexlerae*
14ec6f561033c1df88c4dfe9de7cb89b	*Blautia wexlerae, Blautia sp.* strain Marseille
4114e476e5cb8dfb4087ff72294f3b49	*Blautia wexlerae, Blautia sp.* strain Marseille
b19e4cfacad1b8143a863bbd394eb705	*Blautia faecis, Blautia sp.* strain Marseille
91c177085257768127d1752ad41131ad	*Blautia faecis*
891a7b1d20f52c223cb3f898281ba120	*Blautia sp.* strain Marseille
6a7fd5cbd1a424f24443d9a73c81e579	*Blautia faecis, Blautia sp.* strain Marseille
16a5f67e3dd348b3f871648a32806e9a	*Blautia caecimuris*
59aa338119796a4be404feda2c098d3b	*Blautia caecimuris*
828bdbef43706f3716448118e0b35dbd	*Blautia obeum*
3c1f95d05561a4329d1e6538d08f424c	*Blautia massiliensis*

Supplementary Table 1: Amplicon sequence variants (ASVs) of *Blautia*-related sequences (left) and the potential species, based on blast search (right).
