# Supplementary material for: The gut commensal Blautia maintains colonic mucus function under low-fiber consumption through secretion of short-chain fatty acids
Source: Nat Commun. 2024 Apr 25;15:3502. doi: 10.1038/s41467-024-47594-w (PMC11045866; doi:10.1038/s41467-024-47594-w)
Supplement: Supplementary file 6 — Reporting Summary [file 41467_2024_47594_MOESM6_ESM.pdf]

nature portfolio

Corresponding author(s): Björn O. Schröder

Last updated by author(s): Mar 22, 2024

Reporting Summary

Nature Portfolio wishes to improve the reproducibility of the work that we publish. This form provides structure for consistency and transparency in reporting. For further information on Nature Portfolio policies, see our Editorial Policies and the Editorial Policy Checklist.

Statistics

For all statistical analyses, confirm that the following items are present in the figure legend, table legend, main text, or Methods section.

n/a	Confirmed	
☐	☒	The exact sample size (*n*) for each experimental group/condition, given as a discrete number and unit of measurement
☐	☒	A statement on whether measurements were taken from distinct samples or whether the same sample was measured repeatedly
☐	☒	The statistical test(s) used AND whether they are one- or two-sided<br>*Only common tests should be described solely by name; describe more complex techniques in the Methods section.*
☐	☒	A description of all covariates tested
☐	☒	A description of any assumptions or corrections, such as tests of normality and adjustment for multiple comparisons
☐	☒	A full description of the statistical parameters including central tendency (e.g. means) or other basic estimates (e.g. regression coefficient) AND variation (e.g. standard deviation) or associated estimates of uncertainty (e.g. confidence intervals)
☐	☒	For null hypothesis testing, the test statistic (e.g. *F*, *t*, *r*) with confidence intervals, effect sizes, degrees of freedom and *P* value noted<br>*Give P values as exact values whenever suitable.*
☐	☒	For Bayesian analysis, information on the choice of priors and Markov chain Monte Carlo settings
☐	☒	For hierarchical and complex designs, identification of the appropriate level for tests and full reporting of outcomes
☐	☒	Estimates of effect sizes (e.g. Cohen's *d*, Pearson's *r*), indicating how they were calculated

Our web collection on statistics for biologists contains articles on many of the points above.

Software and code

Policy information about availability of computer code

Data collection	No software was used for data collection.
Data analysis	Amplicons generated during bacterial 16S sequencing were quality filtered using Fastx (http://hannonlab.cshl.edu/fastx_toolkit/index.html) and sequence quality assessed using FastQC (http://www.bioinformatics.babraham.ac.uk/projects/fastqc). Analysis of the human and mouse-derived 16S amplicon sequences was performed using the QIIME2 (version 2022.8) pipeline and R (version 4.1.3) in R Studio (RStudio Team, version 2022.07.2). Sequences were clustered into ASVs using the SILVA classifier (version 138). DADA2 was used to generate the feature table and published R scripts have been used for further data analysis, as described in the methods section. Statistical analyses have been performed with GraphPad Prism (Versions 8/9).The code for 16S data analyses is available at https://doi.org/10.5281/zenodo.10848038.

For manuscripts utilizing custom algorithms or software that are central to the research but not yet described in published literature, software must be made available to editors and reviewers. We strongly encourage code deposition in a community repository (e.g. GitHub). See the Nature Portfolio guidelines for submitting code & software for further information.

Data

Policy information about availability of data

All manuscripts must include a data availability statement. This statement should provide the following information, where applicable:

- Accession codes, unique identifiers, or web links for publicly available datasets
- A description of any restrictions on data availability
- For clinical datasets or third party data, please ensure that the statement adheres to our policy

The human data and stool samples that have been collected for a previously published study are available under restricted access due to data privacy laws and can be requested through the enable cluster (skurk@tum.de). Bacterial 16S rDNA gene sequencing data from the human cohort have been deposited previously in the European Nucleotide Archive under the accession code PRJNA701859 [https://www.ebi.ac.uk/ena/browser/view/PRJNA701859]. Bacterial 16S rDNA data from this study have been deposited under the accession code PRJEB57076 [https://www.ebi.ac.uk/ena/browser/view/PRJEB57076]. Metabolomics data and all raw data generated in this study are provided in the Source Data file.

Research involving human participants, their data, or biological material

Policy information about studies with human participants or human data. See also policy information about sex, gender (identity/presentation), and sexual orientation and race, ethnicity and racism.

Reporting on sex and gender	No human participants were recruited for this study. Stool samples from human donors were obtained from a previously published clinical trial (DRKS00013058). For this present study we ranked the 74 healthy participants (34 men and 40 women, aged between 40-65 years) of the intervention group based on their metabolic improvements and shifts in gut microbiota composition. Details about the ranking and sex distribution of the cohort are described in the manuscript (if relevant), or in the previously published study (Ref 32). The best responders were 4 women and 1 man.
Reporting on race, ethnicity, or other socially relevant groupings	No data on race, ethnicity or other socially relevant groupings were used in this study.
Population characteristics	Population characteristics are described in detail in Brandl et al. (Ref 32): https://doi.org/10.3389/fnut.2022.816299
Recruitment	n/a
Ethics oversight	Ethical Committee of the Faculty of Medicine of the Technical University of Munich (approval no. 201/17S)

Note that full information on the approval of the study protocol must also be provided in the manuscript.

Field-specific reporting

Please select the one below that is the best fit for your research. If you are not sure, read the appropriate sections before making your selection.

☒ Life sciences ☐ Behavioural & social sciences ☐ Ecological, evolutionary & environmental sciences

For a reference copy of the document with all sections, see nature.com/documents/nr-reporting-summary-flat.pdf

Life sciences study design

All studies must disclose on these points even when the disclosure is negative.

Sample size	For all mouse experiments mice were randomly allocated into experimental groups. The investigators were not blinded to allocation during experiments and outcome assessment. Microbiota transplantation from human volunteers into mice to investigate mucus function on viable tissue is a pioneering approach and thus no robust power calculation could be made before the experiments. Based on experience from mouse-to-mouse microbiota transplantation (Ref. 30) we calculated with group averages of 2.2 and 0.9, alpha = 0.05 and a power of 80%, resulting in 8-9 mice per group for the transplantation. For other experiments we pre-determined the number of mice based on previous experiments (Ref 30) with similar bacterial treatments that were able to detect statistically significant differences.
Data exclusions	No data were excluded, except for the mucus growth rate and all related data from one mouse in the HF-Chow group, due to rupture of the colonic tissue during the measurement.
Replication	All recorded data are reported in the manuscript. Biological replicates were used, as indicated in individual data points in all figures.
Randomization	Mice were randomly allocated to either group to best match age and sex. In most cases, mice were housed in groups of 3-4 mice per cage. Analysis of mouse groups was alternated between groups during the termination day to exclude any circadian and/ or reagent bias.<br>Human participants for the stool sample transplantation were selected based on their metabolic parameters and shifts in microbiota compositon from the fiber intervention group. The selection/ranking of the donors is described in the methods section. For other experiments the different groups were always analysed together (e.g. qPCR, MiSeq) to prevent any influence of reagents/equipment. Metabolite stimulation of colonic tissue was carried out from the same mice for treatment and control, and proximal/distal part of the distal colon were alternated between treatments.
Blinding	Mucus measurements of the human FMT, histology analyses and metabolomics were performed blinded. Other experiments were analyzed

by the same researcher that was reponsible for mouse treatment, but randomization as described above prevented any potential bias.

Reporting for specific materials, systems and methods

We require information from authors about some types of materials, experimental systems and methods used in many studies. Here, indicate whether each material, system or method listed is relevant to your study. If you are not sure if a list item applies to your research, read the appropriate section before selecting a response.

Materials & experimental systems

n/a	Involved in the study
☒	☐ Antibodies
☒	☐ Eukaryotic cell lines
☒	☐ Palaeontology and archaeology
☐	☒ Animals and other organisms
☒	☐ Clinical data
☒	☐ Dual use research of concern
☒	☐ Plants

Methods

n/a	Involved in the study
☒	☐ ChIP-seq
☒	☐ Flow cytometry
☒	☐ MRI-based neuroimaging

Animals and other research organisms

Policy information about studies involving animals; ARRIVE guidelines recommended for reporting animal research, and Sex and Gender in Research

Laboratory animals	All mice in this work were C57Bl/6J wild-type mice and groups were age-matched within experiments. The detailed age of the mouse groups is reported in the manuscript and Source Data file for all experiments.
Wild animals	No wild animals were used for this study.
Reporting on sex	Sex was considered for all experiments and mouse groups usually included 50% male/female or close to this ratio, depending on availability. Mucus measurements and/or treatment responses did not differ between sexes. The following numbers were used: Fig 1C-G: 16 males + 16 females; Fig 3C-J: 9 males + 9 females; Fig 4A-E: 5 males + 5 females; Fig 4F-J: 4 males + 8 females; Fig 5A-E: 8 males + 8 females; Fig 6G-O: 35 males + 32 females; Fig S4F-H: 6 males + 6 females; Fig S6D: 4 males + 2 females. Total: 87 males and 86 females = 173 mice.
Field-collected samples	No field-collected samples were used in this study
Ethics oversight	All animal experiments were approved by the local animal ethical committee (Umeå djurförsöksetiska nämnd) with the reference Dnr A14-201).

Note that full information on the approval of the study protocol must also be provided in the manuscript.

Plants

Seed stocks	*Report on the source of all seed stocks or other plant material used. If applicable, state the seed stock centre and catalogue number. If plant specimens were collected from the field, describe the collection location, date and sampling procedures.*
Novel plant genotypes	*Describe the methods by which all novel plant genotypes were produced. This includes those generated by transgenic approaches, gene editing, chemical/radiation-based mutagenesis and hybridization. For transgenic lines, describe the transformation method, the number of independent lines analyzed and the generation upon which experiments were performed. For gene-edited lines, describe the editor used, the endogenous sequence targeted for editing, the targeting guide RNA sequence (if applicable) and how the editor was applied.*
Authentication	*Describe any authentication procedures for each seed stock used or novel genotype generated. Describe any experiments used to assess the effect of a mutation and, where applicable, how potential secondary effects (e.g. second site T-DNA insertions, mosiacism, off-target gene editing) were examined.*
